# Measuring minds: Understanding the mental states of dairy cattle in different management conditions*

**DOI:** 10.3168/jdsc.2024-0712

**Published:** 2025-03-03

**Authors:** Heather W. Neave

**Affiliations:** Department of Animal Sciences, Purdue University, West Lafayette, IN 47907

## Abstract

Minimizing negative and promoting positive emotional states are central to animal welfare. Evaluating these states in dairy cattle under farm conditions is challenging yet vital. This review highlights behavioral (e.g., play, body language), physiological (e.g., heart rate variability, infrared thermography), and cognitive (e.g., judgment bias, flexibility) indicators for assessing emotional states. The selected examples highlight the potential effects of different management conditions on the positive or negative emotional state and cognitive functioning of dairy cattle. Advances in automation have opened avenues for continuous assessment of the mental states of dairy cattle on-farm; this will enable adjustments to management practices to meet individual needs and improve animal welfare.

## Highlights

• Promote positive emotions in dairy cattle, not just minimize negative emotions.
• Explores behavioral, physiological, and cognitive indicators of positive emotions.
• Housing, diet, and enrichment improve emotional and cognitive states in cattle.
• Automation, like sensors and computer vision, enables continuous cattle monitoring.

## Graphical Abstract

**Figure:**
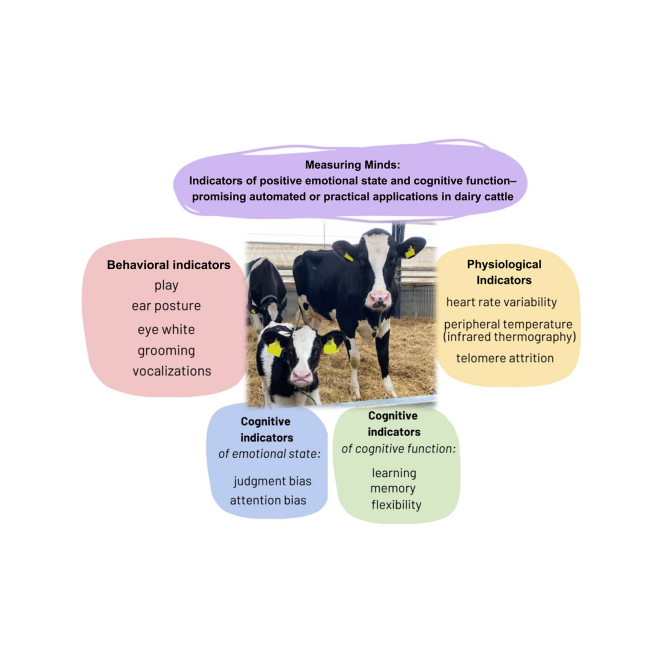
**Summary:** This brief symposium review highlights a selection of ways to assess the mental states of dairy cattle, with a focus on positive emotional states and cognitive function and how they are affected by management practices. It highlights behavioral indicators like play, body postures, and grooming; physiological measures such as heart rate variability and peripheral body temperature; and cognitive tests like judgment and attention bias that evaluate differences in information processing. Tests of cognitive function also reveal learning, memory, and cognitive flexibility as measures of mental state. These methods evaluate how management practices-housing, feeding, and enrichment, for example-positively affect the mental states of dairy calves and cows. Emerging technologies offer exciting possibilities for automated, continuous on-farm monitoring that could enable stakeholders of the dairy industry to adapt practices to support welfare and productivity.

Animal welfare science has traditionally focused on minimizing negative experiences but has shifted toward promoting positive welfare, ensuring animals thrive and experience positive emotional states (Rault et al., 2025). Positive welfare can be supported by enabling essential behaviors (e.g., maternal care) and providing valued resources (e.g., social contact, enrichment; Held and Špinka, 2011), which are expected to lead to positive emotional states. However, the assessment of emotional states in animals is challenging due to their subjective nature, and requires indirect methods validated against known positive or negative situations (Kremer et al., 2020). These contrasting situations should be based on those that animals clearly prefer, find motivating, or avoid, indicating their value and likelihood of being experienced as positive (e.g., motivation for cow to be with her calf; Jensen et al., 2024). Positive situations should also increase comfort behaviors and reduce behavioral or physiological stress indicators (Kremer et al., 2021). Validated emotional state indicators can guide the development of management systems promoting positive welfare in dairy cattle, alongside growing consumer demand for on-farm assessments (Placzek et al., 2020). Of particular relevance is emerging research showing that automation could enable continuous monitoring of emotional states (Neethirajan, 2022). Cognitive functioning, including learning and memory, also provides insight into how management practices affect dairy cattle. Evaluating mental states through cognitive tests of flexibility or memory shows that fostering positive emotional states and improved cognitive functioning can enhance welfare and likely productivity. This brief review highlights behavioral, physiological, and cognitive indicators of positive emotional states in dairy cattle, emphasizing automation and practical applications. Examples illustrate how these indicators assess welfare under different management practices. For deeper insight, readers are directed to reviews of positive welfare indicators in dairy cattle (Keeling et al., 2021), cognitive themes in cattle (Nawroth and R⊘rvang, 2022), and methodologies for assessing emotional states (Ede et al., 2019) and personality (Woodrum Setser et al., 2023).

Of the behavioral indicators of positive emotional states, play has long been regarded as a potential positive welfare indicator, given play is typically only shown when the basic needs of the animal are met (although this is debated in the literature; Held and Špinka, 2011). Play has been used to evaluate possible differences in emotional state between management practices. For example, calves play more when with they have contact with the dam compared with if they are separated at birth (Waiblinger et al., 2020) and play more when pair- versus individually-housed (Duve et al., 2012), although this can also be related to increased space allowance (Jensen et al., 1998). Additionally, calves play more when fed more versus less milk during the preweaning period (e.g., Jensen et al., 2015), and play less during weaning when milk is increasingly restricted, although only 2 studies have used play behavior to evaluate weaning methods that are less stressful (reviewed in Welk et al., 2024). This represents a research gap where a measure of positive emotional state would be informative to evaluate and refine weaning methods in dairy animals. A drawback is that play behavior tends to be rare in adult cattle, and in calves it occurs sporadically, accounting for just a small proportion of a calf's active time (Jensen et al., 2015). Automated recording of play behavior has been validated with leg-based accelerometers in calves (e.g., Luu et al., 2013), making play behavior more accessible and feasible for application on-farm to better understand the impact of management practices on dairy cattle.

Body language could offer insight into the emotional state of dairy cattle; eye white or eye aperture and ear position are the most commonly investigated indicators to date. Studies have examined these indicators under contrasting conditions, such as anticipation of a feed reward (excitement) and frustration (inaccessible or inedible feed; Lambert and Carder, 2017; Lambert and Carder, 2019), or before and after events like cow-calf separation (Sandem and Braastad, 2005) or human stroking (Proctor and Carder, 2014). Generally, lower percentages of visible eye white and relaxed ears at or below the neckline indicated low-arousal positive emotional states (such as relaxation during stroking), and more visible eye white and ears positioned above the neckline or forward indicated higher arousal, whether positive (e.g., food anticipation) or negative (e.g., anxiety during cow-calf separation). This knowledge supports our understanding of how dairy cattle experience different housing and management conditions. For example, Battini et al., (2019) observed that dairy cows at pasture most often had half-closed eyes and relaxed ear postures, unlike during feeding or resting indoors; in fact, these eye and ear positions were almost never observed during an assumed negative situation (human approaching the cow at the feed bunk). Furthermore, de Oliveira and Keeling (2018) described a greater frequency of ears above the neckline during feeding or brushing (presumably positive situations) compared with when queuing for milking. Gómez et al. (2018) reported no change in visible eye white percentage during hoof trimming, a presumed negative emotional state evidenced by greater sympathetic activity and cortisol concentration. These studies highlight the potential of body language to reveal the emotional states of dairy cattle under different management conditions. Recent developments include using computer vision and facial recognition to automate body part position detection, making this measurement method more feasible on-farm (Neethirajan, 2021).

Grooming behavior serves to maintain coat and skin by removing dirt and parasites and can take various forms, including allogrooming, self-grooming, and brushing against objects or provided brushes (Reinhardt et al., 1978). Dairy cows will work for access to a mechanical brush (McConnachie et al., 2018), and calves and heifers will freely approach and use stationary or mechanical brushes, suggesting they find them pleasurable to use (e.g., Zobel et al., 2017; Reyes et al., 2022). Brush use may be a useful and sensitive measure of positive emotional state, given it is a pleasurable activity that declines when experiencing negative situations, such as at parturition and separation from the calf (Lecorps et al., 2021). Thus, measuring changes or differences in brush use could inform how cows perceive different management situations. Recent developments in the automated monitoring of brush use have made this behavior a more feasible indicator of positive emotional states of dairy cows on-farm, such as computer vision using fiducial markers (Sadrzadeh et al., 2024) or ultra-high radio frequency identification of individual cows within proximity of the brush (Falk et al., 2018).

Vocalizations have long been used as an indicator of stress, reflecting negative emotional states in cattle, but there is recent interest in exploring how vocalizations may also be linked with positive emotional states (Laurijs et al., 2021). For instance, low-frequency closed-mouth vocalizations are expressed by the mother cow when her calf is close by (Padilla de la Torre et al., 2015), and low frequency “murmurs” have been recorded from cows while lying and ruminating (Meen et al., 2015). However, high frequency vocalizations can also occur during positive situations, such as during estrus or anticipation of feed (Green et al., 2019), so further validation is needed to determine which acoustic parameters of cattle vocalizations are linked with positive versus negative emotional states. Green et al., (2021) noted that despite the noisy farm environment, empirical call type and temporal vocal features were easy to extract, which lends itself well to potential automation of vocal analysis on-farm. Acoustic sensors attached to the collar or leg of the cow recorded individual vocalizations that could be identified with machine learning algorithms and classified into different vocal types (Shorten, 2023). Once vocal features are validated under positive and negative emotional states, these approaches show the promise of automated acoustic analysis in dairy cattle, which could inform about how cows feel under different management or housing conditions, particularly because such low-frequency murmurs seem to occur during lying and rumination.

Several physiological measures have emerged as possible indicators of positive emotional state, with potential to be automated or continuously assessed on-farm. Heart rate variability was shown to decrease under putatively stressful management practices, such as social isolation (Mandel et al., 2019) or disbudding (Stewart et al., 2009); cows and calves in these studies were interpreted as experiencing negative emotional states. Only a few studies to date have examined how heart rate variability responds under positive conditions; when cows were stroked by a human to presumably induce a positive emotional state, heart rate variability increased as predicted in one study, but not in another (Lange et al., 2020a, Lange et al., 2020b). Peripheral body temperatures (e.g., eye or nasal temperatures) also tend to decrease under stress, due to redirected blood flow to internal body parts during sympathetically-mediated response. For example, maximum eye temperature decreased immediately following disbudding and increased soon after, compared with before the procedure (Stewart et al., 2008). However, other stressors, like dry-off of cows (Franchi et al., 2021) and handling and transport of calves (Lecorps et al., 2018), increased eye temperature after the procedure; these conflicting results may reflect emotional states of pain versus fear (Ede et al., 2019). Assumed positive situations like feeding also slightly raised eye temperature (Gómez et al., 2018). Nasal temperature of cows also increased during assumed positive situations like being stroked by a human (Proctor and Carder, 2015), and decreased during negative situations, like an unexpected change to inedible feed (Proctor and Carder, 2016). These studies collectively suggest that changes in eye or nose temperature may reflect a change in emotional state in dairy cattle, but refinement of experimental design could better distinguish between emotional valence (i.e., positive versus negative). For example, the temperature of the lacrimal caruncle region of the eye of sheep increased during the positive situation (anticipation of feed) but not the negative situation (startle with umbrella; Comin et al., 2024). There is great potential for continuous evaluation of peripheral temperatures in the pen; for example, Lowe et al. (2020) integrated an infrared thermography camera into an automated milk feeder for calves, and validated an algorithm for automatic analysis of calf eye and cheek temperature regions from images. Some examples of precision technologies for measuring physiological attributes are described in calves (Costa et al., 2021) and cattle (Neethirajan, 2017), as well as computer vision approaches for measuring heart rate and ear or eye temperature (Jorquera-Chavez et al., 2019).

Telomere length is emerging as a potential biomarker for tracking lifetime experiences and welfare in animals, including dairy cattle. Telomeres are repetitive DNA sequences and proteins on the ends of chromosomes that shorten with cell division, and shortening is accelerated under oxidative stress and inflammation (Haussmann and Marchetto, 2010). Interestingly, positive experiences in humans can slow or even reverse telomere shortening (Puterman et al., 2015). Thus, telomere length, or its rate of shortening, could be a “molecular record” of positive and negative lifetime experiences in animals (Bateson, 2016). In dairy cattle, heat stress from summer temperatures was shown to accelerate telomere attrition (Seeker et al., 2021). However, there remains a need to evaluate changes in telomere length in putatively positive versus negative situations. Telomere length can be measured from DNA using quantitative PCR techniques (e.g., in blood, saliva, or other bodily fluids such as milk), offering a potential method for assessing cumulative emotional experiences of dairy cattle. One study showed a positive correlation between telomere length in DNA from blood and milk of Agerolese cattle (Iannuzzi et al., 2022), supporting that continuous noninvasive measurements of telomere length may be possible from milk samples.

Emotional state of dairy cattle can also be assessed with a cognitive approach, by testing how cognitive processes (appraisal of stimuli, events, and situations) are affected by emotion (Paul et al., 2005). The judgment bias test evaluates whether animals tend to interpret ambiguous information as more positive or negative (optimism or pessimism) and is now considered a gold standard measure of emotional state in animals (reviewed in Lagisz et al., 2020). Judgment bias has revealed that dairy calves are more pessimistic (i.e., negative emotional state) after disbudding (Neave et al., 2013) or separation from the dam (Daros et al., 2014), and are more optimistic (i.e., positive emotional state) when pair- versus individually-housed (Bučková et al., 2019). However, dairy cows did not show the expected positive judgment bias when housed under presumed positive conditions, such as on pasture (vs. indoors; Crump et al., 2021), indoor housing with environmental enrichment, social group stability and understocking (vs. without these elements; Kremer et al., 2021), and with cow-calf contact (vs. no calf contact; Neave et al., 2024). Although valuable for detecting how certain practices negatively affect calves and cows, judgment bias methods may require refinement to detect low-arousal positive emotional states; extensive training is also required, limiting its on-farm implementation. To date there is no automated version of the task for cattle, but a prototype in laboratory rats could be a starting point, where animals could self-initiate the task in their home pen and interact at any time (Jones et al., 2018).

Attention bias testing assesses whether animals focus on positive or negative stimuli based on their emotional state (Crump et al., 2018). This method showed promise in beef cattle under negative emotional states, where they focused more on a dog threat and were slower to eat after anxiogenic drug administration (Lee et al., 2018), but the opposite effects for positive emotional states were not observed when dairy heifers experienced positive housing conditions (Kremer et al., 2021). In a different attention bias method, cows managed only part time with their calves had a narrowed attentional scope when viewing geometrical hierarchical visual stimuli, consistent with the negative judgment bias results in the same cows (Neave et al., 2023). Although these approaches offer insights into emotional states in dairy cows, their training demands remain high, requiring practical refinements for broader application.

The mental states of dairy cattle can also be evaluated in terms of how management practices promote or impair cognitive function, which are interpreted as better or worse for the animals' welfare. For instance, calves that were pair-housed or group-housed with their dams instead of individually-housed performed better on reversal learning of a visual go/no-go task and object recognition task (Gaillard et al., 2014; Meagher et al., 2015), suggesting improved memory and cognitive flexibility when calves have some level of social contact. Physical enrichment of calf housing pens also enhanced object recognition (Zhang et al., 2022). Dietary enrichment, such as hay provision or feeding more milk through a teat, improved T-maze learning and adaptability when the route changed (e.g., Horvath et al., 2017). After weaning, calves that performed well in reversal tasks integrated more easily into new social groups, highlighting that cognitive performance and social adaptability may be linked (Horvath and Miller-Cushon, 2018). Conversely, restrictive feeding practices, such as during weaning, impaired memory in an adapted hole-board task requiring spatial exploration (Lecorps et al., 2023). These studies exemplify how housing and dietary practices can mentally affect dairy calves in terms of their cognitive development and adaptability in early life. To date there are no studies reporting the cognitive functioning of adult dairy cattle as a consequence of current or early-life housing and management practices; based on this work in calves, it is predicted that improved cognitive function early in life will lead to more flexible, adaptable and resilient cows when facing management challenges later in life.

In summary, this review has highlighted methods to assess dairy cattle mental states, focusing on positive emotions and automated on-farm applications. Key behavioral and physiological indicators include play, body language, grooming, vocalizations, heart rate variability, infrared thermography, and telomere attrition. Cognitive tests, such as learning, memory, and cognitive bias assessments, evaluate emotional states and the effects of management practices. Advancements in automation and continuous-monitoring technologies, like wearable sensors and computer vision, can accelerate and deepen our understanding of the mental states of dairy cattle, enabling tailored management practices that improve their welfare and productivity.
